# Health Professionals’ Experiences with Treatment Engagement Among Immigrants with Co-occurring Substance Use- and Mental Health Disorders in Norway

**DOI:** 10.1177/11782218211028667

**Published:** 2021-07-06

**Authors:** Prabhjot Kour, Lars Lien, Bernadette Kumar, Ole Martin Nordaunet, Stian Biong, Henning Pettersen

**Affiliations:** 1 Norwegian National Advisory Unit on Concurrent Substance Abuse and Mental Health Disorders (NK-ROP), Innlandet Hospital Trust, and University of South-Eastern Norway, Norway; 2 Norwegian National Advisory Unit on Concurrent Substance Abuse and Mental Health Disorders (NK-ROP), Innlandet Hospital Trust, and Faculty of Health and Social Sciences, Norway University of Applied Sciences, Norway; 3 Norwegian Institute of Public Health, Oslo, Norway; 4 Drammen Municipal Mental Health and Drug Addiction Services, Drammen, Norway; 5 University of South-Eastern Norway, Norway

**Keywords:** Co-occurring substance use and mental health disorders, immigrants, health professionals, mental health and addiction services, qualitative methods, lived experiences, Norway

## Abstract

Immigrants face barriers in seeking and accessing mental health and addiction services. Health professionals are crucial in providing and promoting healthcare and it is important to understand their experiences in order to enhance the access of mental healthcare. The aim of this paper is to explore and describe health professionals’ experiences with treatment engagement among immigrants with co-occurring substance use disorders (SUD) and mental health disorders (MHD) in Norwegian mental health and addiction services. Within a collaborative approach, 3 focus group interviews were conducted with health professionals, who had provided various mental health and addiction care services to immigrants with co-occurring SUD and MHD. The focus group interviews were transcribed verbatim and analyzed using systematic text condensation. The analysis resulted in 5 main categories: (1) difficulties due to language barriers, (2) difficulties due to lack of culturally competent services, (3) difficulties due to social factors, (4) being curious and flexible improves the user-provider relationship, and (5) increasing access to mental health and addiction services. This study provides an enhanced understanding of how health professionals’ experienced treatment engagement among immigrants with co-occurring SUD and MHD in the Norwegian context. Implications of the findings for clinical practice and future research are discussed.

## Introduction

Various studies have investigated racial/ethnic disparities in immigrants’ access to mental health services.^1-3^ Research in Norway,^1^ Sweden,^4^ the Netherlands,^5^ and Finland^3^ has documented the underutilization of specialist mental health services by immigrants in comparison to the host population. Although the Norwegian healthcare system is grounded in principles of equity and solidarity,^6^ and immigrants with a residence permit are entitled to similar health services as the host population,^7^ immigrants face barriers in accessing these services.^8^ Barriers to accessing mental health services are well documented in the literature; these include lack of information about available services, transport and language difficulties, difficulties in navigating the healthcare system, lack of trust, perceived stigma, different understandings of mental health, and thus different perceived needs for care.^9-11^ A systematic review reported that barriers to treatment or to maintaining care included communication difficulties with the healthcare provider, lack of culturally adapted services, and inability of the provider to understand different cultural meanings of mental health disorders (MHDs).^12^ In addition to these barriers, mental health professionals’ stereotyping and biased or discriminatory treatment when dealing with immigrants have also been reported.^13,14^ Further, Mladovsky et al^15^ have pointed out that immigrants with substance use disorders (SUDs) and MHDs are at high risk of neglect due to lack of existing healthcare policies and insufficient funding to target specific areas of immigrants’ mental healthcare. This situation may be aggravated by health professionals’ limited knowledge of immigrants’ backgrounds, leading to immigrants’ dissatisfaction with the services.^16-18^

The rate of immigration to Norway has considerably increased over recent decades; immigrants form a growing proportion of the Norwegian population,^19,20^ which leads to an ethnically diverse society.^21^ The share of immigrants in Norway’s total population is 18.2%, while 10.8% of the population are immigrants from middle- and lower-income countries.^19^ The most common countries of origin of this 10.8% are Somalia, Pakistan, Iraq, Syria, and Eritrea.^19^ The most common foreign language spoken is English, among others are Urdu, Somali, and Persian. Most health professionals speak English, but all patients in Norway have the right to an interpreter, who is paid by the government, not by the patient.^22^ Studies show that immigrants may be at high risk of developing MHDs due to various pre- and post-migration factors.^21,23^ Typically, MHDs and substance use present with a high degree of co-occurrence,^24-26^ accompanied by poor quality of life.^27^ People diagnosed with co-occurring SUD and MHD often face challenges in getting support for tailored and integrated treatment.^28^ Moreover, several factors influence their utilization of health services^20^ and may lead to lower treatment engagement.^18,29-31^ These factors may include their expectations based on experiences in their home countries, lack of culturally tailored services, and previous negative and discriminatory experiences with health services in the host country.^32^

Understanding the process of treatment engagement among immigrants with co-occurring SUD and MHD through health professionals’ experiences can provide a basis for the treatment. Engagement is understood as the process of establishing a mutually collaborative, trustful, and respectful helping relationship.^33^ Stewart theorizes that the success of engagement can be determined by the quality of the care and relationship built between service users and health professionals during the treatment process,^34^ Integrating the principles of “person-centered healthcare” (PCH) in mental health and addiction services has been shown to enhance the quality of relationships and engagement, with improved outcomes.^35^ PCH is understood as health services respecting the uniqueness of individuals by focusing on their values, beliefs, desires, and wishes, regardless of their age, gender, social status, faith, financial situation, ethnicity, and cultural background.^36^ PCH has emerged as a cornerstone of effective SUD treatment^37,38^ and is highlighted in Norwegian national guidelines for SUD treatment.^39^ This implies that PCH may improve treatment engagement among immigrants.

In addition to PCH, a cultural competence approach in healthcare has been seen to reduce racial/ethnic disparities (such as threshold for seeking care, ability to communicate symptoms comprehensibly to health professionals, expectations for care, and adherence to treatment), while also improving the quality of care for immigrants.^40^ A culturally competent healthcare system is understood as one that “acknowledges and incorporates—at all levels—the importance of culture, the assessment of cross-cultural relations, vigilance toward the dynamics that result from cultural differences, expansion of cultural knowledge, and adaptation of services to meet culturally unique needs.”^40^ Cultural competence among mental health professionals working in diverse environments is increasingly recognized as an essential skillset. Various approaches have been developed to enhance the cultural competence of mental health professionals, such as ethnic matching of user and provider, and providers may modify their mode of interaction with users, develop culturally adapted interventions, or offer interventions drawn from the user’s own cultural traditions.^41^ Training materials and resources have been made available for these professionals, such as textbooks and a specific curriculum and guidelines for training in cultural psychiatry.^42^ Additionally, PCH and cultural competence approaches are believed to have the same core features, and hold promise for improving the quality of healthcare for individuals^43^ and thus enhancing immigrants’ treatment engagement.

In former research on treatment experiences of immigrants living with co-occurring SUD and MHD, some participants narrated various reasons for their lower treatment engagement in mental health and addiction services in Norway; these included a lack of culturally competent staff.^18^ Health professionals are crucial in providing and promoting healthcare and thus there is a need to understand their experiences of treatment engagement among immigrants with co-occurring SUD and MHD, which to best of our knowledge has not been previously studied in Norway.

The aim of this paper is to explore the health professionals’ experiences with treatment engagement among immigrants with co-occurring SUD and MHD.

## Methods

An explorative and descriptive qualitative research design was implemented, with a collaborative approach. Collaborative research is considered highly relevant to the research process and clinical practice, and can improve the evidence base used to inform how services are provided.^44^ The kind of collaborative approach used in this study is “mainstream interest in user involvement in research,” where the focus was on seeking and including the views of service users in the research process.^44^ Hence, drawing on the literature on collaborative research, a competency group of 3 persons was established. Aiming to include different groups affected by the study,^45^ 2 members were previous users with lived experience of having co-occurring SUD and MHD, and 1 was a relative of one of the users included. All 3 were immigrants and had an understanding of both their original local context and the Norwegian context. The group worked as advisors to the research team in all stages of the study: planning the study, developing the focus group (FG) interview guide, recruitment approaches, analysis and understanding the results in a local context, as well as dissemination.

### Context

Norwegian mental health and addiction services are mostly divided into 2 levels: primary healthcare^46^ (based on social, health and welfare legislation, run by municipalities) and secondary healthcare^47^ (based on specialist healthcare legislation, run by the state). The specialist services include various polyclinics and psychiatric centers, including opioid substitute treatment facilities, multidisciplinary specialized addiction treatment centers, district psychiatric centers, and hybrid solutions in partnership with primary healthcare, namely (flexible) assertive community treatment (FACT/ACT) teams.

Specialized MHD/SUD treatment centers provide diagnosis and treatment, including drug therapy, psychiatric and family therapy with the aim of helping individuals to desist from substance use, and improve their functioning in relation to health, work, and family. Primary healthcare services (municipalities) in Norway adhere to many of the recommendations for locally based healthcare. The recommendations emphasize that services aim to deliver a comprehensive healthcare services spanning from harm-reductive strategies that is needle exchange, opioid treatment and low-threshold services, to a strengths based recovery oriented approach with focus on activities, talking therapies and groups, and the need for a cooperative healthcare system between specialist and primary healthcare such as stated in the guideline provided by the Norwegian Directorate of Health.^48^ In addition, introduction programs for new immigrants form part of primary healthcare. Introduction programs are designed for newly arrived immigrants; here, they are given basic skills in the Norwegian language, insight into Norwegian culture and society, and preparation for work or education.^49^ Moreover, the municipality in question offers mental health services based on recovery-oriented principles and feedback-informed treatment as stated in the guideline provided by the Norwegian Directorate of Health.^48^ The FACT team is multidisciplinary, consisting of psychiatrists, nurses, psychologists, social workers, and peer workers with user experiences. FACT uses an assertive outreach approach to assist persons with mental health and substance use problems and their local communities, and cooperates with specialist and primary healthcare, in terms of reducing hospitalizations, and enhancing the person’s well-being in the local community with individual follow-up care in the areas of work, family, leisure, and housing.^50^

### Recruitment

This study forms part of a larger project with an exploratory and descriptive aim, which examines, on the one hand, the experiences of immigrants with co-occurring SUD and MHD with coping^16^ and treatment in Norwegian mental health and addiction services,^18^ and on the other hand, health professionals’ experiences of these factors. It was decided to conduct the project in the 2 Norwegian cities, Oslo and Drammen, with the highest proportion of immigrants, 33% and 29% respectively.^51^ Because of the large immigrant population in these cities,^51^ health professionals there were expected to have the most contact with immigrants and experience of providing various mental health and addiction care services.

Recruitment was initiated by emailing and calling the leaders of various primary and secondary mental health and addiction services with access to potential immigrant patients with co-occurring SUD and MHD. These leaders received detailed information about the research project. They then sent the information to their team members and only those health professionals who were willing to participate in the study were included. The recruitment was strategic, being aimed at health professionals with experience of providing a variety of mental health and addiction care services to immigrants with co-occurring SUD and MHD in Norway. These were a specialized MHD/SUD treatment center, primary (municipal) healthcare, and a flexible assertive community treatment (FACT) team.

### Participants

The study included 19 participants, 12 females and 7 males, divided into 3 FGs. Their ages ranged from 28 to 65 years, while their experience of treating immigrants with co-occurring SUD and MHD ranged from 1 to 25 years. The participants had different professional backgrounds (Appendix A, Table A1), including 1 psychiatrist, 3 psychologists, 4 specialist nurses, 6 general nurses, and 5 social workers. Sixteen of the 19 participants were ethnic Norwegians and 3 were of immigrant origin. FG 1 consisted of 7 health professionals from the specialized MHD/SUD treatment team. FG 2 was composed of 8 health professionals from primary healthcare, and FG 3 comprised 4 health professionals working in a FACT team.

### Data collection

Prior to data collection, an FG interview guide, consisting of open-ended thematic questions about experiences of providing various mental health and addiction care services to immigrants with co-occurring SUD and MHD was created and agreed upon by all authors and the members of the competency group. Table A2 (Appendix B) shows the interview guide with main questions, which all had follow-up and probing questions.

The data were collected through FG interviews, as the method was considered to be well suited to enquire about the participants’ experiences, perceptions, desired goals and difficulties, and could provide a deeper understanding of their attitudes.^52^ In addition, FG interviews assist in exploring phenomena and experiences that are incompletely understood and sensitive issues that may not be captured by the prevailing literature or expert opinion.^53^ Three FG interviews were conducted with health professionals between November 2019 and February 2020. The FG interviews were conducted at the participants’ workplaces, as per their convenience and working hours. The FG interviews lasted between 60 and 80 minutes and were audio recorded. They were led by moderators and the first author. Two different moderators were used: FG 1 was conducted by the first moderator, and FGs 2 and 3 by the second moderator. The first moderator could not continue for FGs 2 and 3 because of commitments elsewhere, and a second moderator was therefore used for the remaining FG interviews. The second moderator was also the interpreter, who transcribed all 3 FG interviews and translated them into English. The first author acted as an observer at all 3 FG interviews, concentrating on group dynamics, and noting down thoughts that arose when following the dialog. At the end of each FG interview, the moderator and first author shared their reflections and later discussed them with all authors and the members of the competency group.

### Data analysis

Within the aim of the study, the analysis was combined inductive-deductive, that is, PCH and cultural competence were applied as theoretical background and inspiration, while inductive in a sense that the participants’ descriptions were the point of departure. The FG interviews were transcribed and translated to English by the interpreter. Systematic text condensation (STC),^54^ was employed to analyze these interview transcripts. Further, STC is an explorative and descriptive method which aims at thematic cross-case analysis, and which also maintains methodological rigor and enables intersubjectivity, feasibility, and reflexivity.^54^ STC is a stepwise procedure that encompasses identification of recurring initial codes and themes relevant to the study aim. In step 1, a general impression was formed by reading all the transcripts, which led to the formation of initial themes. In the second step, after a systematic review of the transcripts, meaning units were identified and sorted into code groups. In the third step, subgroups were formed from the code groups with their meaning units. The next step was to form artificial quotations by reducing the meaning units under each subgroup. The final step was to develop the analytic text and descriptions from the artificial quotations. The analytic text was reconceptualized by returning to the complete transcripts and reflecting on whether each illustrative quotation still reflected the original content. This was done in order to validate the analytic texts. Lastly, the analytic texts were supported by quotes, which are presented in the “Results” section. In each step, all the co-authors were consulted, and discussions took place. In the final step, the team of experts by experience was consulted to provide an understanding of the results within the local context they represented.

### Ethical statement

The study was conducted in accordance with the Declaration of Helsinki and was approved by the Norwegian Centre for Research Data (Project No. 59707). The participation was completely voluntary. All FG participants signed informed consent and received an information letter about the project prior to the group interviews. Confidential details of the participants were deleted from the data in order to maintain their anonymity. The informed consent contained information on the study, including the aim and purpose of the research. It also mentioned the selection of participants, voluntary participation, duration, potential risks and benefits, confidentiality, anonymity, right to withdraw, and dissemination of results. The contact information of the first author was provided in case the participants had concerns or questions after the FG interviews. The moderator and interpreter signed a confidentiality declaration prior to the interviews.

## Results

The analysis yielded 5 main categories describing health professionals’ experiences of treatment engagement among immigrants with co-occurring SUD and MHD. These categories were: (1) Difficulties due to language barriers, (2) Difficulties due to lack of culturally competent services, (3) Difficulties due to social factors, (4) Being curious and flexible improves the user-provider relationship, and (5) Increasing access to mental health and addiction services. Each category is described below with reference to the empirical data and numbers used to identify the participants follow each excerpt.

### Difficulties due to language barriers

When treating immigrants with co-occurring SUD and MHD participants stated that both they and the immigrant patients faced difficulties due to the language barrier. These difficulties were often experienced as frustrations due to miscommunication and were described as a constraining factor in diagnosis and treatment. Further, some participants mentioned that group therapy sessions were among standard treatments for patients with co-occurring SUD and MHD, but that many immigrants were excluded from group therapy because they could not speak and understand Norwegian, and if included, they changed the dynamics of group sessions by disturbing others and reducing spontaneity. Additionally, a few participants highlighted difficulties experienced due to language barriers and miscommunication in one-to-one counseling sessions, where effective communication was considered vital in engaging patients with co-occurring SUD and MHD.

*And the difficulty has been to understand how good their language skills are, if we need an interpreter or not. It has also been difficult to know if they should get 24-hour treatment and if they understand enough [Norwegian] to participate in group sessions. Because almost all treatment we have here is based on group therapy. . . .And we would not be able to have an interpreter here 24 hours a day so. . . They [non-Norwegian speakers] are in a way excluded from that. . .* (P2, FG-1)

Further, a few participants described difficulties in using interpreters with immigrant service users with a poor command of Norwegian. They mentioned that sometimes the interpreters were not competent enough to understand issues related to co-occurring SUD and MHD, which led to frustrations for both the patients and the participants themselves. Also, sometimes the use of different interpreters in different sessions with the same patient led to unsuccessful communication. Seeing a new interpreter at every new session was described as inconvenient and often created miscommunication between the parties. Moreover, a few participants mentioned difficulty in obtaining interpretation services since these were not cost-effective in Norway, resulting in lower use of interpreters, and often only in crucial situations.

*So I have had patients that went through 5-6 different interpreters, or at least five different interpreters. And that is not appropriate because there is the confidentiality and the trust that are necessary if they are to share things. I see that as a difficulty. . ..* (P5, FG-1)

Having to work with immigrants with poor Norwegian language skills was perceived as difficult as it required extra effort and extra hours of work in their busy schedule. One participant described it in the following way:

*And the other thing that you realize about patients from other cultures or with bad Norwegian. . . it is a busy working day and to understand that someone doesn’t speak Norwegian is almost extra work and we find it difficult. An extra. . .extra work. . ..* (P4, FG-3)

### Difficulties due to lack of culturally competent services

Most of the participants described difficulties due to the lack of culturally competent services in treatment. Further, it was stated that not knowing the person’s cultural background often caused misunderstandings of what was socially and culturally acceptable behavior. Some participants also linked this to their feeling of helplessness in not knowing on how to approach these patients in treatment. This led to difficulty in developing a trusting relationship and when the cultural expectations of these patients were not met, they tended to discontinue the treatment. Further, participants talked about the lack of available resources such as official courses, training or guidelines on cultural sensitivity in treatment programs. This gave them limited opportunity to acquire knowledge and skills in cultural competence.

*We have very few resources, knowledge and skills about cultural competence . . . Also, I think, when it comes to how. . . what do we do? I mean, how, what do I do? What should I do if I meet an immigrant patient, how do I get things started?* (P1, FG-3)

Some participants also mentioned that there are very few immigrant health professionals in Norwegian mental health and addiction services. One participant said that immigrant patients often ask for health professionals of immigrant origin and it causes difficulties when they do not get access to these.

*I have been working with this for over 20 years at many different places. I have met one. . . one psychologist who was from a non-Western country. And. . . two or three nurses. . . I don’t know why, but there are too few non-Western immigrants working in the Norwegian mental healthcare and addiction services. . .* (P5, FG-1)

In addition, a few participants who work in introduction programs mentioned that there is little focus on mental health and substance use in the programs. This means that such problems continue undetected and develop in severity before the program counselor notices them. In such cases, the problems can lead to new problems and require more effort to address and treat.

### Difficulties due to social factors

Participants mentioned various social factors that discourage immigrants from seeking and engaging in treatment. Predominant factors were poor integration, isolation, and marginalization. Some participants also mentioned that immigrants often live in parallel societies, where they have very little contact with mainstream society, in addition to the stigma attached to SUDs and MHDs.

*I think that immigrants have problems with participating. They do not come out of their contexts. Not very willing to be integrated. Living in parallel worlds. Like, they are not well integrated and it seems like many cultures live in parallel societies.* (P4, FG-2)

Further, participants highlighted immigrants’ poor financial and living conditions. They described how the combination of co-occurring SUD and MHD, stigma attached to them, and poor coping with the transition from their home country to Norway with few supportive contacts is a potential barrier to the treatment engagement process. In addition, there is no local immigrant network that can help to improve treatment engagement.

*. . ..I mean, my experience is that when you take, typical areas of immigrant housing. And you see a kindergarten where all the children are dark skinned. . . And then there is housing for addicts, where there is a lot of substance use, just next door. And the heroin addicts or khat-chewing old people are sitting there, and there is the mosque right next door, and there is a place where a lot of garbage gets thrown.. . . and stuff like that. I mean, that proximity of non-existing maintenance, drugs, crime. . . That would not be tolerated in areas where Norwegians live. . . So. . . I think it plays a part. That immigrants feel like they can’t demand the same. . . That it is in a way a double standard. That you don’t have the right to be treated the same way or have the same attention. . .* (P2, FG-3)

Some of the participants mentioned that having poor Norwegian language skills also leads to social exclusion. The participants further experienced that this hampers the possibility to seek knowledge about the available treatment options and leads to a poor understanding of how the Norwegian mental health and addiction services work.

*And it was very challenging, as I recall, when it came to the. . . immigrants who were badly integrated, did not speak Norwegian and didn’t have any formal knowledge or understanding of the society. . .. it was very challenging. . .*. (P3, FG-2)

### Being curious and flexible improves the user-provider relationship

Some participants described how being curious and flexible while treating immigrants with co-occurring SUD and MHD had improved their relationships with them. They also mentioned that when they had met their expectations based on the cultural context and acknowledged that cultural differences existed when tailoring individual treatment, this had engaged immigrants and enabled them to complete treatment.

*Being open-minded and curious,. . .. I have a patient who has some difficulties and I go and I read about that, you see? When they grew up in a country that I don’t entirely understand. So then I go and read up on that a bit. . .. It’s possible to read. . . read a book about that but it’s because they grew up in a country, take up the topic. . .* (P1, FG-3)

A few participants mentioned that allowing immigrant patients to practice their faith while they are in treatment enhanced the engagement process.

*We try to accommodate. . . all the religions and. . .When we want to build up [a relationship], we could have a diversity room, a place where they can pray or. . . or do what they want.* (P6, FG-1)

A few participants found that their relationship improved by having a welcoming attitude with an interest in how persons of immigrant origin function, coupled with communicative skills of being creative, and using non-verbal communication. One participant mentioned that the ability to find flexible solutions to address communication problems, along with frequent and close follow-up, has improved their relationships.

*Being aware that they can have, not necessarily, but they can have a different understanding of the sickness, another understanding of why I am sick and what is the sickness. . . to be a fellow human being, to be curious, be interested. . ..* (P1, FG-1)

### Increasing access to mental health and addiction services

A few participants showed a general concern about the possible lack of primary mental healthcare accessible to immigrants with co-occurring SUD and MHD. Further, 1 participant mentioned that providing practical help to immigrants, such as a mental health nurse who already has a relationship with them accompanying them to their doctor, has facilitated the participation and engagement process. Moreover, some participants stated that using professional and competent interpreters who have cultural competence can aid in building trust in therapy sessions.

*We had a psychiatric nurse that worked with us who had a very important influence. She worked hard to: I mean, when I sit there as program consultant and counselor and. . . and I see that there is something here, something that doesn’t. . . fit. So. . . I have to come in. . . in a position where the participant I want to talk to talks to our psychiatric nurse. And then she would carefully assess their health, mental and physical health and then she would also accompany us to the person’s doctor, and we started there to see if there is a need for, what kind of needs there are. . .* (P5, FG-2)

Additionally, a few participants also stated that increasing the accessibility of mental health and addiction services by making them ambulatory has improved treatment engagement. Addressing the complex challenges faced by immigrants, such as service providers going to their homes when they cannot come to the treatment centers, or meeting in parks, as per their convenience, with close follow-up, helping them with their everyday work and providing injections, has helped to build a trusting relationship.

*I think, when it comes to what we can do; for us working in FACT, I think we are very ambulatory, go home to people, follow them up closely, we help them with everything from hanging up the curtains to giving them injections. To be able to get, to get trust, a relationship, that’s what keeps our work going.* (P3, FG-3)

## Discussion

This study illuminates health professionals’ experiences with treatment engagement among immigrants with co-occurring SUD and MHD in Norway. Five main categories were identified which we classified into 2 major sections as barriers and facilitators to treatment engagement and have discussed the results in these 2 sections. The first section consisted of barriers that reduced treatment engagement, which were difficulties due to language barriers, difficulties due to a lack of culturally competent services, and difficulties due to social factors. The second section consisted of facilitators that enhanced treatment engagement, which were being curious and flexible to improve the user-provider relationship, and increasing accessibility of mental health and addiction services.

### Barriers to treatment engagement

Participants described how difficulties due to language barriers led to miscommunication, limiting diagnosis and treatment, and problems in group therapy sessions, which lowered treatment engagement among immigrants with co-occurring SUD and MHD. Effective communication between health professionals and users is a key factor in mental healthcare, as diagnosis is based on verbal communication rather than on objective physical examinations.^55^ A study conducted in 16 major European cities reported that language difficulties were a major barrier in assessing symptoms, making a diagnosis, and developing a trusting relationship with immigrant patients,^56^ which resonates with our findings. In addition, a few studies have reported that insufficient language skills among immigrants act as a barrier to accessing specific mental health treatments such as psychotherapy.^57,58^ This is similar to our findings, where participants stated that immigrants faced difficulties in joining group therapy sessions conducted in Norwegian, which were among the standard treatment approaches in their services. This can be seen as a lack of PCH and a cultural competence approach in the treatment, where services seem less accessible for immigrants with co-occurring SUD and MHD, representing a barrier to treatment engagement.

Further, participants experienced using interpreters often led to miscommunication and lower treatment engagement because of incompetent interpreters who did not understand the issues of co-occurring SUD and MHD. This has also been reported in previous studies conducted among mental health professionals providing treatment to immigrants.^40,58-60^ In addition, language difficulties and unskilled interpreters have been reported to be frustrating for both health professionals and immigrant patients, with negative effects on treatment alliances and establishing trust,^59,60^ as reflected in our participants’ experiences, leading to poor treatment engagement. Moreover, a survey conducted among psychotherapists reported that 43% of them refused to treat immigrant patients due to language disparities,^61^ which concurs with our findings where some participants perceived treating immigrants as involving extra effort and extra hours. Further, the narrative of extra efforts and extra work can be seen as a discriminatory element in the treatment process, where immigrant patients were not given the attention and care they needed, which can be correlated with our previous findings.^16,18^ Furthermore, some of the participants mentioned the use of interpreter services as neither cost-effective nor commonly used in their work experience. This is in contrast to the guidelines of the Norwegian Directorate of Health, which state that patients with limited knowledge of Norwegian are entitled to an interpreter in their preferred language.^62^ Additionally, all these findings indicate the lack of PCH and a cultural competence approach, which ultimately leads to a lack of individually tailored services for immigrants with co-occurring SUD and MHD and hence acts as a barrier to treatment engagement.

Another barrier that shaped the treatment engagement of immigrants with co-occurring SUD and MHD, as reflected in the participants’ experiences, was difficulties faced due to lack of cultural competence in treatment programs. Participants described how the lack of cultural competence caused misunderstandings of what is socially and culturally acceptable behavior for immigrant patients and hence decreased treatment engagement, in line with previous studies.^18,55-57^ These misunderstandings could also be understood in terms of immigrants’ narratives of experiencing lack of connection and lack of individually tailored services while in treatment for co-occurring SUD and MHD.^18^ This was exacerbated by the lack of official courses and relevant resources for health professionals to learn about cultural competence in mental health settings.^57,63^ Moreover, health professionals who feel unequipped to deal with comprehensive needs arising during interactions with immigrant patients have different understandings of mental illness and treatment,^57,63^ which resonates with our findings. Further, a lack of diversity among healthcare workers and a need for immigrant professionals in mental health and addiction services are well documented in the literature.^55,57,58^ This concurs with our findings and was seen in our sample, where only 3 of 19 health professionals were of immigrant origin. In addition, Betancourt et al^40^ argue that lack of diversity in the leadership and staff of healthcare organizations may result in poorly designed structural policies, procedures and delivery systems to meet the needs of immigrant patients, thus representing a barrier to their treatment engagement.

In addition, some participants mentioned difficulties due to social factors that reduced treatment engagement among immigrants with co-occurring SUD and MHD. Cultural interpretation of mental health needs, stigma attached to SUDs and MHDs and experiences of discrimination and social exclusion often cause difficulties in engaging in treatment.^18,55,57^ This is exacerbated by poor knowledge of available help in mental health and addiction services,^59^ as reflected in our findings, where participants mentioned lower treatment engagement by immigrants because of poor integration and living in parallel societies with few contacts in mainstream Norwegian society. Some studies have also reported that socioeconomic factors including poor living conditions, low income levels, and unemployment act as barriers to utilizing mental health and substance use treatment,^59,64^ in line with our participants’ experiences. Additionally, in our previous study,^16^ the immigrants’ narrative of “living a double life,” “cultural clash,” and “racism and cultural stigma” acted as barriers to seeking or continuing treatment, which concurs with the participants’ experiences of facing difficulties due to social factors.

### Facilitators to treatment engagement

To facilitate the adequate use of available treatment and to ensure lower drop-out rates, it is critical to understand the barriers.^65^ PCH^35^ and a cultural competence approach^40^ have been shown to facilitate treatment engagement. According to the framework suggested by Saha et al,^43^ both PCH and cultural competence have overlapping core values that work at the interpersonal level (between health professionals and users) and the health system level. At the core of PCH and cultural competence at the interpersonal level is health professionals’ ability to see the patient as a unique person, to maintain an unconditional positive attitude, to build effective rapport, and to explore the patient’s beliefs, values, and meaning of illness, in order to find a common ground for treatment plans, which is also aided by understanding the meaning and importance of culture, and the effective use of interpreters.^43^ This is similar to our findings where participants described how being curious to learn about different cultural contexts, being flexible to provide individually tailored treatment, and being creative to find solutions to address communication problems, have all improved their therapeutic relationships and hence facilitated treatment engagement among immigrants with co-occurring SUD and MHD. Previous studies on healthcare professionals’ perspectives have also shed light on the importance of cultural competence in mental health treatment for immigrants.^55,56^

Furthermore, at system level, PCH and cultural competence, an emphasis on enhancing health professionals’ accessibility, a diverse workforce that reflects the minority population and partnering with communities in setting priorities and planning, are all associated with improved treatment outcomes.^43^ This further promotes equity for minority groups, who tend be disadvantaged in terms of seeking care and in treatment engagement.^43^ The systemic level could be understood in terms of the participants’ experiences where they mentioned that making mental health and addiction services accessible to immigrants with co-occurring SUD and MHD facilitates treatment engagement, including increased accessibility of mental health professionals, immigrant health professionals, the use of professional interpreters, and the integration of mental health and addiction services with primary services, which is consistent with previous studies.^55,57^ Further, participants from the FACT team mentioned that having ambulatory services tailored to individual immigrant patients’ needs has facilitated their process of treatment engagement, which aligns with the principles of PCH and cultural competence at the systemic level. These interventions may help to reduce racial/ethnic disparities in healthcare and truly provide quality healthcare^40^ for immigrants with co-occurring SUD and MHD, who are overshadowed by the majority in treatment engagement.

Lastly, on the one hand, from a PCH and cultural competence perspective, it is important to adapt services to meet the needs and preferences of immigrants with co-occurring SUD and MHD in treatment, to increase the accessibility of health services in immigrant communities, including outreach and home visits, and to ensure that health information material is tailored to immigrants’ needs, preferred language and health literacy.^43^ On the other hand, from a policy perspective, it is crucial to identify the causes of unmet needs.^66^ Here we would point out the need to understand factors that may be responsible for the unmet needs of immigrants and the barriers preventing health professionals from providing tailored treatment. The participants pointed out that some of these factors are the language barrier, limited cultural sensitivity, and a lack of resources to enable professionals to acquire cultural competence. Some such factors were also narrated by immigrants with co-occurring SUD and MHD, including lack of individually tailored services, lack of connection between health professionals and immigrants, stigma and discriminatory experiences with healthcare, and distrust of the system, in our previous studies.^16,18^ Previous studies have suggested some strategies to address these issues and to improve mental health and addiction service delivery, such as highlighting the importance of culturally appropriate services by acknowledging users’ needs and preferences, and encouraging users to voice their own explanations of health, worries, and expectations.^67,68^

## Limitations and strengths

The present study provides an overview of health professionals’ experiences with treatment engagement among immigrants with co-occurring SUD and MHD in Norway. The results are based on the experiences of the participants and their relevance beyond the local context may be discussed. Nevertheless, exploring subjective experience involves a focus on the insights and meaning of the participants, which may be transferred^69^ to other people and other contexts. Additionally, these insights are believed to be of relevance for future research in the field of migrant healthcare, where there is a paucity of research. Further, the collaboration with the competency group in all stages of the study, from writing the protocol, preparing the study, analyzing the data to compiling the results, has enhanced the credibility^69^ of our study.

Conducting a study with an explorative and descriptive qualitative research design of this kind requires the researcher to avoid approaching the FG interviews with too fixed questions and pre-understandings. Therefore, the first author focused on letting the participants’ voices be heard and refrained from any early interpretations and judgment of the meaning of their experiences. However, the first author’s background as a medical doctor and immigrant herself may have influenced the analysis. Selective bracketing of pre-understanding was sought during analysis, even though complete bracketing is seen as impossible. To this end, the competency group was involved in the process of analysis to offer validation and reflexivity and may have addressed this potential limitation.^70^ Additionally, all the co-authors had several discussions about the analysis process.

This study has an important methodological limitation, namely that it was only possible to recruit health professionals from 3 different services and those who were willing to participate. This may be considered as selection bias during recruitment and we may not have reached health professionals with more experience of treating immigrants with co-occurring SUD and MHD. However, with the limited time frame of this study, this was the only feasible approach. A longer-term strategy could have made it easier to reach health professionals from other Norwegian mental health and addiction services. Another limitation was lack of in-depth discussion on how participants described “immigrants.” However, most participants briefly described “immigrants,” as persons who were born or whose parents were born outside Norway. Here, they referred to the standard definition of immigrants used in Norway and provided by the national agency, Statistics Norway (SSB). One participant referred to “immigrants” as persons with a multicultural background in his clinical practice.

Further, one can discuss the methodological challenge of recruiting from health services with different treatment approaches in the same study. However, few immigrants seek mental health and addiction services in Norway,^1^ thus it was expected that few health professionals would have had experience of treating immigrants. As such, we were not focused on context-specific treatment; our goal was to explore the experiences of health professionals working in Norwegian mental health and addiction services, in order to understand immigrants’ treatment engagement. Our purpose was to find immigrants’ treatment engaging behavior in different services, according to different health professionals, including psychologists, psychiatrists, nurses, and social workers. In addition, this could be linked to our previous study, where immigrants voiced their reasons for their lower treatment engagement and lack of satisfaction when their needs remained unmet.^18^ Hence, conducting this research on health professionals’ experiences may have helped to provide a multifaceted view of the barriers to treatment engagement among immigrant patients.

## Conclusion

Health professionals’ experiences with treatment engagement among immigrants with co-occurring SUD and MHD were described and explored, which shed light on both barriers and facilitators to treatment. Difficulties due to language barriers, social factors, and the lack of culturally competent services were described as important ongoing barriers and hence led to lower treatment engagement. However, health professionals’ ability to be curious and flexible, and to make mental health and addiction services accessible to immigrant patients, facilitated treatment, and thus improved treatment engagement. The findings of this study indicate an increased need for competent and professional interpreters who have knowledge about mental health and substance use problems, in order to overcome language barriers. A culturally competent approach is clearly lacking; therefore, we suggest placing greater emphasis on strategies that provide person-centered and culturally competent services. The cultural competence of the healthcare professionals providing treatment to immigrants must be enhanced and cannot depend on their personal interest only. Hence, we suggest that providing resources to teach healthcare professionals about diverse cultural backgrounds is paramount to improve treatment engagement among immigrants with co-occurring SUD and MHD. Future research on how to enhance understanding of these barriers in the context of immigration, and intervention studies to enhance PCH and cultural competence in healthcare settings are recommended.

## Appendix A

**Table A1.** Participants’ profession and distribution in the focus groups.

Professional background	Focus group 1: specialized MHD/SUD treatment center	Focus group 2: primary healthcare services	Focus group 3 FACT team	Total
Psychiatrist			1	1
Psychologist	2		1	3
Specialist nurse	1	2	1	4
General nurse	4	1	1	6
Social worker		5		5
Total	7	8	4	19

## Appendix B

**Table A2.** Interview guide.

Questions
1. Can you describe your experiences of providing treatment services to immigrant patients with co-occurring SUD and MHD?
2. Can you describe what in your experience promoted treatment in these patients?
3. Can you describe the barriers to treatment in your experience?
4. Can you describe trust building strategies, if you have experience of using any, and how these worked with these patients?
5. Can you describe any experience you have of using culturally competent strategies with these patients?
6. Would you describe your sources of knowledge of culturally competent treatment, if any?
7. Can you describe to what extent treatment needs are adapted to individual immigrant patients in your experience?
